# Inhibition of NET Release Fails to Reduce Adipose Tissue Inflammation in Mice

**DOI:** 10.1371/journal.pone.0163922

**Published:** 2016-10-04

**Authors:** Quinte Braster, Carlos Silvestre Roig, Helene Hartwig, Linda Beckers, Myrthe den Toom, Yvonne Döring, Mat J. Daemen, Esther Lutgens, Oliver Soehnlein

**Affiliations:** 1 Department of Pathology, Academic Medical Center (AMC), Amsterdam, the Netherlands; 2 Department of Medical Biochemistry, Academic Medical Center (AMC), Amsterdam, the Netherlands; 3 Institute for Cardiovascular Prevention (IPEK), LMU Munich, Munich, Germany; 4 DZHK (German Centre for Cardiovascular Research), partner site Munich Heart Alliance, Munich, Germany; Technische Universitat Dresden, GERMANY

## Abstract

Obesity-associated diseases such as Type 2 diabetes, liver disease and cardiovascular diseases are profoundly mediated by low-grade chronic inflammation of the adipose tissue. Recently, the importance of neutrophils and neutrophil-derived myeloperoxidase and neutrophil elastase on the induction of insulin resistance has been established. Since neutrophil elastase and myeloperoxidase are critically involved in the release of neutrophil extracellular traps (NETs), we here hypothesized that NETs may be relevant to early adipose tissue inflammation. Thus, we tested the effect of the Peptidyl Arginine Deiminase 4 inhibitor Cl-amidine, a compound preventing histone citrullination and subsequent NET release, in a mouse model of adipose tissue inflammation. C57BL6 mice received a 60% high fat diet for 10 weeks and were treated with either Cl-amidine or vehicle. Flow cytometry of adipose tissue and liver, immunohistological analysis and glucose and insulin tolerance tests were performed to determine the effect of the treatment and diet. Although high fat diet feeding induced insulin resistance no significant effect was observed between the treatment groups. In addition no effect was found in leukocyte infiltration and activation in the adipose tissue and liver. Therefore we concluded that inhibition of neutrophil extracellular trap formation may have no clinical relevance for early obesity-mediated pathogenesis of the adipose tissue and liver.

## Introduction

Obesity-associated diseases such as Type 2 diabetes, liver disease and cardiovascular diseases are profoundly mediated by low-grade chronic inflammation of the adipose tissue (AT). During obesity, expansion of AT-resident leukocytes, the recruitment of immune cells and the switch from anti- to pro-inflammatory environment results in insulin resistance and other metabolic complications [1, 2]. Upon initiation of obesity, neutrophils are one of the first immune cells to infiltrate the AT [3]. These granulocytes are involved in the induction of insulin resistance via secretion of their granule proteins Neutrophil Elastase (NE) and myeloperoxidase (MPO) [4, 5]. NE was suggested to induce insulin resistance by degradation of insulin receptor substrate 1 and enhancing leukocyte influx involving toll like receptor 4 signaling, while MPO reduces expression of insulin receptor β and enhances chemokine expression resulting in influx of inflammatory cells. However, neutrophil degranulation is not the sole mechanism to deliver granule proteins to the extracellular space. In fact, presentation of neutrophil granule proteins within neutrophil extracellular traps (NET) is an alternative route to display granule proteins in high concentrations. NETs are neutrophil-derived structures composed of a DNA scaffold decorated with nuclear, cytosolic, and granular proteins. While release of NETs (NETosis) was initially described as an anti-microbial defense mechanism [6], it has more recently become clear that NETosis plays an important role in the development of chronic inflammatory diseases such as atherosclerosis and Systemic Lupus Erythematosus [7, 8]. While the precise mechanism underlying NET formation is not fully understood, it has been shown that MPO and NE are critically important during NET release and that neutrophils deficient in these proteins are unable to produce NETs [9–11]. Since neutrophil-derived NE and MPO are both connected to the induction of insulin resistance as well as release of NETs, we here hypothesized that NETosis links early neutrophil influx with AT inflammation by stimulating inflammation and mediating leukocyte influx. Therefore we aimed to study the effect of NETosis during the early stages of obesity-mediated pathogenesis. This was done by inhibition of NETosis during the first ten weeks of obesity by the inhibitor Cl-amidine.

## Material and Methods

### Mice

The animals were housed according to institutional regulations and the animal procedures were approved by the Committee on the Ethics of Animal Experiments of the University of Amsterdam and the Animal Research Institute (ARIA), Amsterdam.

Six weeks old C57BL6/J mice were purchased from Charles River. At eight weeks of age they received a 60% High-Fat-Diet (HFD) (D12492 (I) mod. 60%/kcal energy derived from fat [Lard]; SNIFF) for 10 weeks and a daily subcutaneous injection of either Cl-amidine (10 mg/kg bodyweight; Essen Scientific) or the vehicle (0.9% NaCl). This treatment regimen was adopted from previous studies employing prolonged Cl-amidine treatment in models of chronic inflammation [7, 8, 12]. Following ten weeks of injections the mice were euthanized by ketamine/xylazine overdose, blood was removed by heart puncture and the mice were perfused with 20 mL ice cold PBS supplemented with 5 mM EDTA. Subsequently the adipose tissue and liver were removed for paraffin embedding and freeze storage.

### Glucose Tolerance Test/Insulin Tolerance Test

Three days before the start and end of the HFD the glucose and insulin tolerance was determined by glucose and insulin tolerance tests (GTT/ITT). Briefly, mice were starved for 12 (GTT) or 4 (ITT) hours after which they received an intraperitoneal injection of 1 mg/g bodyweight glucose (GTT) or 1.1 mU/g bodyweight human insulin (Sigma-Aldrich) (ITT). Subsequently the blood glucose was measured by glucometer (Bayer Contour) at indicated time points.

### In vitro NET release assay

Isolated neutrophils were seeded in a 96 well plate and stimulated with either medium (HBSS; Life Technologies) or Phorbol 12-myristate 13-acetate (100 nM; PMA; Sigma-Aldrich) in presence or absence of Cl-amidine (1 mM). DNA release was assessed by presence of 5 μM SYTOX green nuclear acid stain (Life Technologies) and measured in a Victor plate reader (Wallac) over a set period.

### Immunofluorescence and Histochemistry

Paraffin sections of white adipose tissue (5μm thickness) were stained with rat anti mouse Ly6G (1:100; BD Biosciences) and rabbit anti mouse Cathelicidin (1:100; Innovagen). Subsequently, samples were incubated with Alexa fluor 647 donkey anti rabbit (1:500; Invitrogen), donkey anti rat 549 (1:500; Invitrogen), and the DNA-binding dye DAPI (Invitrogen). Co-localization of these markers was determined by confocal microscopy (SP-8 X, Leica). In addition paraffin sections of white adipose tissue were stained with hematoxylin and eosin. Adipocyte size was determined of 100 adipocytes per mouse by use of ImageJ.

### Plasma cholesterol

Plasma levels of cholesterol were measured by CHOD-PAP assay (Roche) according to the manufacturer’s description.

### Flow Cytrometric Analysis

White adipose tissue (500 mg/mouse) was digested by Liberase (0.25 mg/ml; Roche) at 37°C for 45 minutes. Subsequently the digest was filtered through a 70 μM nylon mesh to obtain a single cell suspension. Liver resident immune cells were isolated by smashing 500 mg liver through a 70 μM nylon mesh following a 36% percoll separation (20 min, 800 g at RT). Leukocyte populations were identified by flow cytometry using antibodies to CD45, CD11b, Ly6G, F4/80, CD11c, MHCII, CD3, CD4, CD8, NK1.1, and B220 (all eBioscience).

### Protein isolation from AT and Dot Blot

AT was homogenized in a ceramic mortar in presence of 500 μL isolation medium (50mM Tris, 150 mM NaCl, 0,2mM EDTA, and protease inhibitors (cOmplete mini, Roche)) and 1875 μL chloroform:methanol mixture (1:2). Homogenate was transferred to a 15 mL tube on ice, mixing every 5 minutes for 15 min. Subsequently, 625 μL chloroform and 625 μL water were added after which the mixture was centrifuged (800 g, 5 min, 4°C). The white “middle” band was collected, resuspended in 100 μL isolation medium, and used for protein determination (Pierce TM BCA protein assay kit, ThermoFisher). 2 μg protein was spotted on two Nitrocellulose membranes (0,2 μm, BIO-RAD), dried (30 min, 37°C), and stained for either rabbit anti-Histone H3 (1:500, Abcam) or rabbit anti-Histone H3-Citrulline (R2+R8+R17) (1:500, Abcam), followed by donkey anti rabbit HRP (1:1000, ThermoFisher). Blots were visualized by ChemiDoc MP system using Clarity Western ECL substrate and quantified by Immage lab 5.0 using round volume tools (all BIO-RAD).

### RNA isolation and qPCR

RNA was isolated from AT by Trizol (Ambion, Life Technologies) according to manufacturer’s instruction and cDNA was prepared by QuantiTect Reverse Transcription Kit (Qiagen). qPCR was performed on the Applied Biosystems HD 7900, using QuantiNova SYBR Green RT-PCR Kit 500 (Qiagen) and primers against TNFα, IL-6, IL-10 and S18 Ribosomal (Qiagen).

### Statistical analysis

All data is presented as mean ± SEM. Significance was determined by prism 5 (graphpad) making use of either the student-t-test, Mann-Whitney, or two-way ANOVA corrected for multiple comparisons by Sidak's, Dunnett's or Tukey's test. P < 0.05 was considered as a significant difference.

## Results

**Fig 1:**
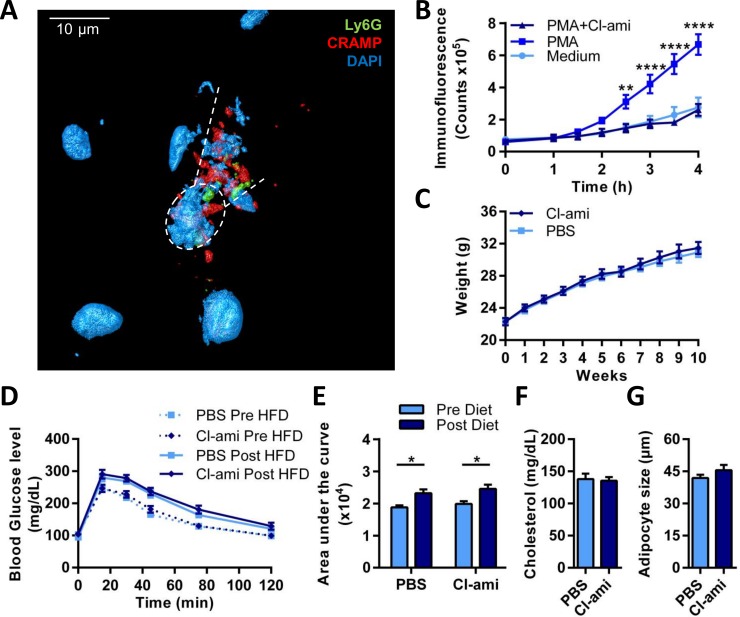
Cl-amidine treatment does not affect metabolic changes associated with high fat diet feeding. **A)** 3D reconstruction of a NET in obese adipose tissue of HFD fed C57Bl6 mice imaged by immunofluorescence confocal microscopy. Co-localisation of the markers Ly6G (green), cathelicidin (red), and dapi (blue) is shown. Dotted circle shows a NET-releasing neutrophil between the dotted lines. **B)** Isolated neutrophils were incubated with medium, 100 nM PMA, or 100 nM PMA and 1 mM Cl-amidine (Cl-ami). NETosis was measured with the extracellular DNA dye Sytox green over a period of 4 hours (n = 7). **C-G)** Mice receiving a 60% HFD for ten weeks were treated with either Cl-amidine (n = 10; 10 mg/kg; s.c.; daily) or vehicle (PBS) (n = 10; s.c.; daily). **C)** The mice were weighted weekly. **D)** Prior (pre-HFD) and after 10 weeks of the HFD (Post-HFD) a glucose tolerance test was performed. The mice were fasted for 12 hours after which they received a subcutaneous injection of 1 mg glucose per g mice. Blood glucose levels were measured at different time points. **E)** Area under the curve was calculated from the GTT graphs Fig 1D. **F)** Plasma levels of cholesterol were measured by CHOD-PAP (Roche). **G)** Haematoxylin/eosin staining was performed on paraffin sections of EpAT and the diameter of 100 adipocytes per mice was calculated. Data is represented as mean ± SEM, *p = 0.05, **p = 0.01, ***p = 0.001, ****p = 0.0001, n = 10 for each group unless stated otherwise.

To assess the presence of NETs in the AT of obese mice we stained white AT of high fat diet fed C57Bl6 mice. Confocal microscopy following immuno-fluorescent staining for the neutrophil surface marker Ly6G, the granule protein cathelicidin, and DNA, revealed co-localization of these makers indicative of NETs (**Fig 1A**). One critical step during NET formation is the chromatin decondensation which is associated with histone citrullination, a process catalyzed by the enzyme Peptidyl Arginine Deiminase 4 (PAD4). Treatment with Cl-amidine, a specific PAD inhibitor, reduces NET release and disease severity in various disease models [7, 8, 12]. Prior to in vivo inhibition of NET release, we assessed the ability of the PAD4 inhibitor Cl-amidine to prevent NET release in vitro. Isolated neutrophils were stimulated with either medium or 100 nM PMA in presence or absence of 1 mM Cl-amidine. In these experiments, Cl-amidine fully prevented the formation of NETs (**Fig 1B**).

Subsequently twenty C57Bl6 mice received a HFD for ten weeks. Neutrophils have been shown to be recruited to the adipose tissue shortly after initiation of high fat diet feeding [3, 4]. However, we here chose to study a ten week time point to capture initial functional changes post neutrophil recruitment. Ten mice received a daily subcutaneous injection of 10 mg/kg Cl-amidine while the other ten mice were injected daily with vehicle only. HFD induced a significant increase in bodyweight with no difference between the treatment groups (p = 0.57) (**Fig 1C**). To study glucose tolerance and insulin resistance, we performed glucose and insulin tolerance tests before and after HFD feeding. A significant decrease in glucose tolerance and a gain in insulin resistance was observed upon HFD feeding, but no difference was detected between the treatment groups (**Fig 1D and 1E** and **S1A Fig**). Bodyweight, weights of liver, epididymal AT, subcutaneous AT, brown AT and spleen, as well as cholesterol levels did not differ between the treatments group (**Fig 1F** and **S1B Fig**). Hematoxylin and eosin staining showed no difference in adipocyte size (**Fig 1G**) and liver steatosis (Data not shown). To confirm the effectiveness of Cl-amidine during the experiment a dot blot was performed on epididymal AT of lean, obese vehicle (PBS) injected, and obese Cl-amidine injected mice. Quantification of the dot blot showed an increased citrullination status of histone H3 in obese PBS treated mice compared to lean mice, which was not observed in the obese Cl-amidine treated mice (**S1C Fig**).

**Fig 2:**
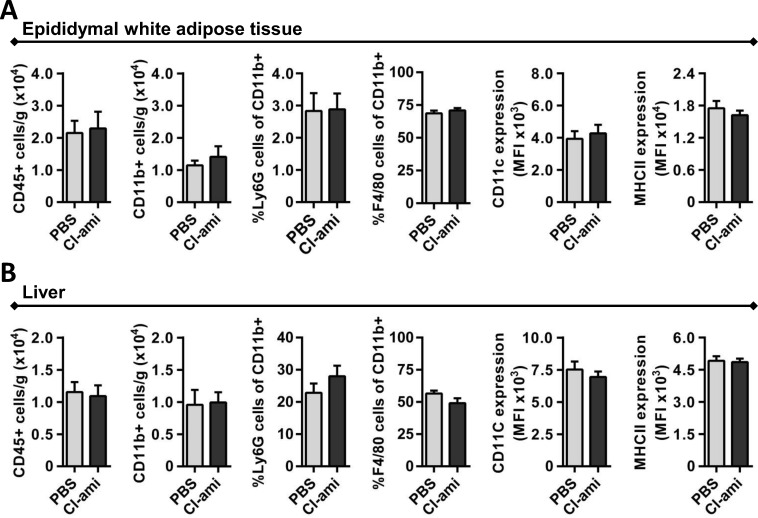
Effect of Cl-amidine treatment on immune cell accumulation and activation in adipose tissue and liver. **A)** White adipose tissue was digested by 0.25 mg/mL Liberase to isolate the resident immune cells. Flow cytometry analysis was performed for leukocytes (CD45^+^), myeloid cells (CD45^+^/CD11b^+^), neutrophils (CD45^+^/CD11b^+^/Ly6G^+^), macrophages (CD45^+^/CD11b^+^/F4/80^+^) and expression of macrophage pro-inflammatory markers CD11c and MHCII on macrophages. **B)** Similar flow cytometry was performed on liver resident immune cells. Data is represented as mean ± SEM, n = 10 for each group unless stated otherwise.

With Cl-amidine having no effects on the metabolic status, we further assessed possible effects on inflammation. One important step during AT inflammation is the recruitment of leukocytes [13]. To study accumulation and activation of neutrophils and macrophages the white AT was digested with Liberase. Single cell suspensions obtained from epididymal and subcutaneous AT were stained with specific antibodies and the data were acquired by flow cytometry (**Fig 2A and S2 Fig**). No significant differences between the treatment groups were observed in the number of CD45^+^ leukocytes or CD11b^+^ myeloid cells. Similarly, no changes in the fraction of Ly6G^+^ neutrophils and F4/80^+^ macrophages were detected (**Fig 2A**). Since we found no differences in the number of cells we investigated the phenotype of the macrophage by mean fluorescence intensity of CD11c and MHCII. However, in these analyses no changes were observed, which was consistent with qPCR analyses performed on AT-derived RNA (**S1D Fig**). Finally, no differences were found in the number of cytotoxic T cells, T helper cells, natural killer cells and B cells (identified by CD3, CD4, CD8, NK1.1, B220 staining; all antibodies eBioscience) (Data not shown). Since the Cl-amidine treatment did not affect the AT resident leukocytes we additionally studied leukocyte accumulation in the liver. Consistent with the AT data no differences were observed in the number of CD45^+^ leukocytes and CD11b^+^ myeloid cells, as well as the fractions Ly6G^+^ neutrophils and F4/80^+^ macrophages. Also the activation status of macrophages was not affected by Cl-amidine treatment (**Fig 2B**).

## Discussion

Mechanisms underlying obesity-associated diseases like type-two diabetes and cardiovascular diseases have been intensively investigated over the recent years. In case of cardiovascular diseases, plasma markers of NET release were found to correlate with severe coronary atherosclerosis and inhibition of NETosis resulted in a reduced disease development in mice [8, 14]. Since the pathogenesis of cardiovascular diseases and obesity are closely connected and essentially mediated by leukocyte influx and activation, we assumed that inhibition of NET release may be beneficial in a mouse model of adipose tissue inflammation. In addition, neutrophil-derived NE and MPO are linked to both the induction of insulin resistance and the release of NETs. We therefore hypothesized that NETosis links early neutrophil influx with subsequent AT inflammation. However inhibition of NETosis in obese mice with the PAD4 inhibitor Cl-amidine, a compound successfully used in several studies to prevent NET-associated pathogenesis [7, 8, 15–17], did not exhibit beneficial effects despite the presence of NETs in adipose tissue. Differences were found neither in metabolic parameters like mice weight, organs weight, adipocyte size, plasma cholesterol levels, insulin resistance and glucose tolerance nor in inflammatory parameters like leukocyte influx and activation in the adipose tissue and liver. This result was not due to the inability of Cl-amidine to inhibit NETosis, since Cl-amidine was shown to be able to inhibit NETosis and similar concentrations were used as in previous studies [7, 8]. Therefore it seems that inhibition of NETosis by Cl-amidine treatment does not reduce the inflammation reaction in the liver and adipose tissue resulting from high fat diet feeding. Although we find no relevant role of NETosis in the onset of obesity and insulin resistance, recent studies indicate roles in advanced obesity and diabetes. It was shown that type-2-diabetic patients have more factors linked to NETs like dsDNA and elastase in their plasma possibly due to the fact that their neutrophils are more prone to undergo NETosis mediated by high glucose levels [18, 19]. In addition, NET-releasing neutrophils impaired wound healing, which is of critical importance to patients with Type II diabetes [18]. Thus, these findings suggest a potential therapeutic role for NET inhibition in advanced obesity and associated pathologies but not in the onset of obesity.

## Supporting Information

S1 Fig Cl-amidine treatment does not affect glucose tolerance, organ weights, and inflammation parameters. **A)** Prior (pre-HFD) and after 10 weeks of the HFD (Post-HFD) an insulin tolerance test was performed. The mice were fasted for 4 hours after which they received a subcutaneous injection of 1.1 mU glucose per g mice. Blood glucose levels were measured at different time points. **B)** After euthanasia of the mice the liver, spleen, epididymal adipose tissue (EpAT), subcutaneous adipose tissue (ScAT) and brown adipose tissue (BAT) were weighted. **C)** Dot Blot was performed on AT-derived protein for Hisone H3 and Histone H3 Citrulline (n = 5). The ratio H3 Citrulline over Histon H3 was quantified. **D)** qPCR was performed on AT derived RNA for TNFα, IL-6, IL-10 and S18 Ribosome (N = 3). ΔΔCT was calculated against S18 Ribosome. Data is represented as mean ± SEM, *p = 0.05, **p = 0.01, ***p = 0.001, ****p = 0.0001, n = 10 for each group unless stated otherwise.
(TIFF)

S2 Fig Effect of Cl-amidine treatment on resident immune cells in Subcutaneous Adipose Tissue. Subcutaneous adipose tissue was digested by 0.25 mg/mL Liberase to isolate the resident immune cells. Flow cytometry analysis was performed for leukocytes (CD45^+^), myeloid cells (CD45^+^/CD11b^+^), neutrophils (CD45^+^/CD11b^+^/Ly6G^+^), macrophages (CD45^+^/CD11b^+^/F4/80^+^) and expression of macrophage pro-inflammatory markers CD11c and MHCII on macrophages. Data is represented as mean ± SEM, n = 10 for each group unless stated otherwise.
(TIFF)
