# Supplementary figures and images for: Inhibition of NET Release Fails to Reduce Adipose Tissue Inflammation in Mice
Source: PLoS One. 2016 Oct 4;11(10):e0163922. doi: 10.1371/journal.pone.0163922 (PMC5049774; doi:10.1371/journal.pone.0163922)

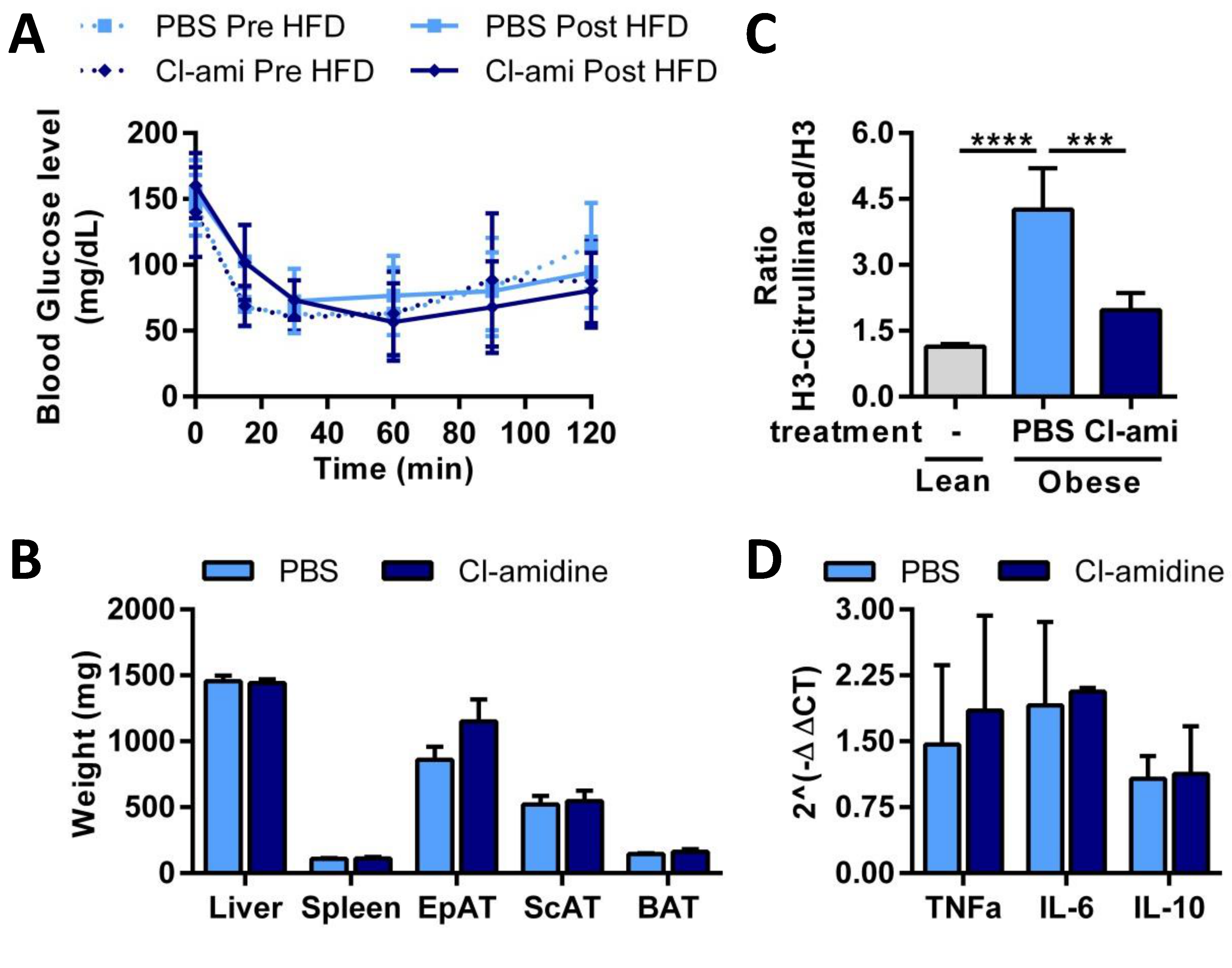

Supplement: S1 Fig — A) Prior (pre-HFD) and after 10 weeks of the HFD (Post-HFD) an insulin tolerance test was performed. The mice were fasted for 4 hours after which they received a subcutaneous injection of 1.1 mU glucose per g mice. Blood glucose levels were measured at different time points. B) After euthanasia of the mice the liver, spleen, epididymal adipose tissue (EpAT), subcutaneous adipose tissue (ScAT) and brown adipose tissue (BAT) were weighted. C) Dot Blot was performed on AT-derived protein for Hisone H3 and Histone H3 Citrulline (n = 5). The ratio H3 Citrulline over Histon H3 was quantified. D) qPCR was performed on AT derived RNA for TNFα, IL-6, IL-10 and S18 Ribosome (N = 3). ΔΔCT was calculated against S18 Ribosome. Data is represented as mean ± SEM, *p = 0.05, **p = 0.01, ***p = 0.001, ****p = 0.0001, n = 10 for each group unless stated otherwise. (TIFF) [file pone.0163922.s001.tiff]

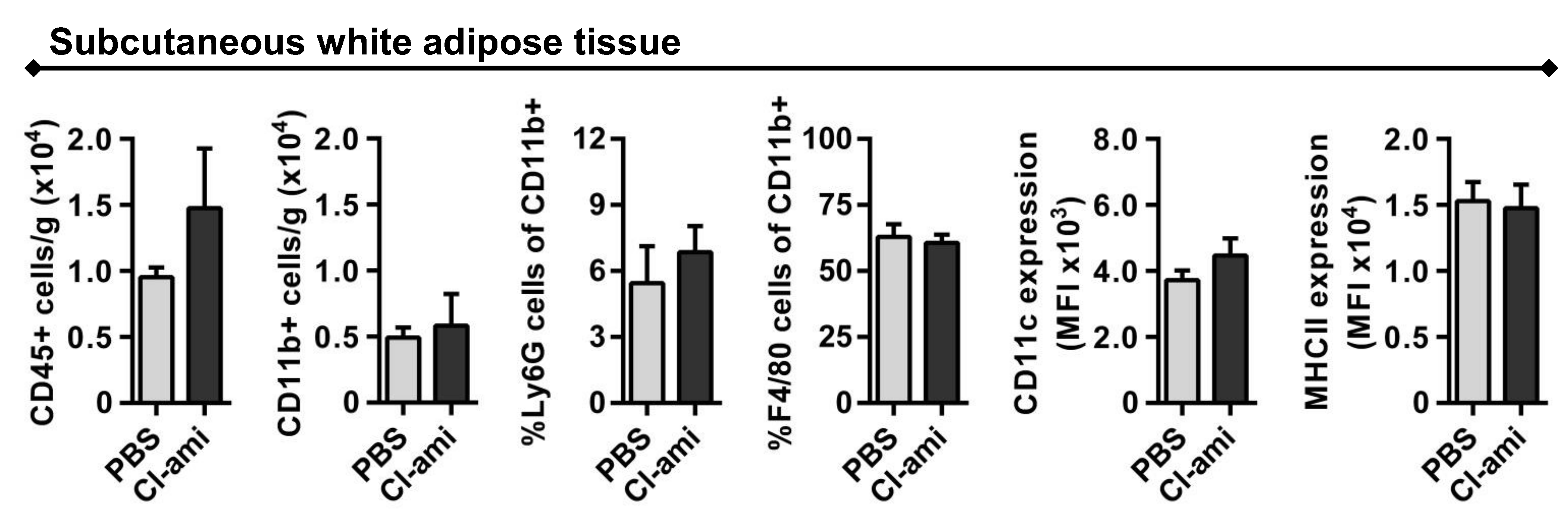

Supplement: S2 Fig — Subcutaneous adipose tissue was digested by 0.25 mg/mL Liberase to isolate the resident immune cells. Flow cytometry analysis was performed for leukocytes (CD45+), myeloid cells (CD45+/CD11b+), neutrophils (CD45+/CD11b+/Ly6G+), macrophages (CD45+/CD11b+/F4/80+) and expression of macrophage pro-inflammatory markers CD11c and MHCII on macrophages. Data is represented as mean ± SEM, n = 10 for each group unless stated otherwise. (TIFF) [file pone.0163922.s002.tiff]
